# Discovery of Cryoprotective Activity in Human Genome-Derived Intrinsically Disordered Proteins

**DOI:** 10.3390/ijms19020401

**Published:** 2018-01-30

**Authors:** Naoki Matsuo, Natsuko Goda, Kana Shimizu, Satoshi Fukuchi, Motonori Ota, Hidekazu Hiroaki

**Affiliations:** 1 Laboratory of Structural Molecular Pharmacology, Graduate School of Pharmaceutical Sciences, Nagoya University, Furo-cho, Chikusa-ku, Nagoya, Aichi 464-8601, Japan; naoki.matsuo@hb.nagase.co.jp (N.M.); tenno.natsuko@f.mbox.nagoya-u.ac.jp (N.G.); 2 Department of Computer Science and Communications Engineering, Waseda University, Okubo, Shinjuku-ku, Tokyo 169-8555, Japan; shimizu.kana@waseda.jp; 3 Faculty of Engineering, Maebashi Institute of Technology, Maebashi 371-0816, Japan; sfukuchi@maebashi-it.ac.jp; 4 Graduate School of Informatics, Nagoya University, Furo-cho, Chikusa-ku, Nagoya, Aichi 464-8601, Japan; mota@i.nagoya-u.ac.jp; 5 The Structural Biology Research Center and Division of Biological Science, Graduate School of Science, Nagoya University, Furo-cho, Chikusa-ku, Nagoya, Aichi 464-8601, Japan

**Keywords:** intrinsically disordered proteins, dehydrin, cryoprotection, lyophilization protection, molecular shields, biomedical application

## Abstract

Intrinsically disordered proteins (IDPs) are an emerging phenomenon. They may have a high degree of flexibility in their polypeptide chains, which lack a stable 3D structure. Although several biological functions of IDPs have been proposed, their general function is not known. The only finding related to their function is the genetically conserved YSK_2_ motif present in plant dehydrins. These proteins were shown to be IDPs with the YSK_2_ motif serving as a core region for the dehydrins’ cryoprotective activity. Here we examined the cryoprotective activity of randomly selected IDPs toward the model enzyme lactate dehydrogenase (LDH). All five IDPs that were examined were in the range of 35–45 amino acid residues in length and were equally potent at a concentration of 50 μg/mL, whereas folded proteins, the PSD-95/Dlg/ZO-1 (PDZ) domain, and lysozymes had no potency. We further examined their cryoprotective activity toward glutathione *S*-transferase as an example of the other enzyme, and toward enhanced green fluorescent protein as a non-enzyme protein example. We further examined the lyophilization protective activity of the peptides toward LDH, which revealed that some IDPs showed a higher activity than that of bovine serum albumin (BSA). Based on these observations, we propose that cryoprotection is a general feature of IDPs. Our findings may become a clue to various industrial applications of IDPs in the future.

## 1. Introduction

The concept of intrinsically disordered proteins (IDPs), also known as natively unstructured proteins [1], has been drawing attention, particularly during the past decade [2,3,4,5,6,7]. After the completion of several genome projects for different organisms, the researchers found a large number of IDPs, as well as intrinsically disordered regions (IDRs). These proteins do not seem to adopt any unique and solid conformation under physiological conditions. For a long time, it has been believed that nascent proteins must undergo a folding process to acquire structure for their appropriate biological functions. The strong correlation between amino acid sequence, three-dimensional (3D) structure, and function has been referred to as “the central dogma of structural biology” [1]. Thus, the biological importance of IDPs in terms of their specific function should be further examined.

The most distinctive feature of IDPs/IDRs is that they lack tertiary structures with stable compact folding under physiological conditions. This biophysical feature of disordered regions is closely related to their amino acid sequences, which is different from ordered regions [8]. Classically, disordered regions are enriched in disorder-promoting amino acids, predominantly including hydrophilic or charged amino acids (A, R, G, Q, S, P, E, and K). In addition, these disordered regions do not include the order-promoting hydrophobic or aromatic amino acids (W, C, F, I, Y, V, L, and N) [9]. As a result, IDPs are characterized as having low averaged hydrophobicity with net charges [10]. Based on these observations, predictions of IDPs from their primary amino acid sequences have been successfully achieved through bioinformatics, which is coping with a rapidly increasing number of genome sequences. Examples of prediction tools in this category include DisEMBL [11], GlobPlot [12], PONDR VSL1 [13], DISOPRED [14], DISpro [15], and VSL2 [16]. We also developed different IDP predictors, DICHOT [17,18], and a series of web applications named POODLE [19,20,21]. In this study, we designed our experiment based mainly on the POODLE suites, which automatically integrate the results from three different programs (POODLE-S, POODLE-L, and POODLE-W) according to the lengths of the IDPs.

Using bioinformatics analyses of IDPs, we could thus identify general features related to molecular evolution: (1) the disordered regions may have a faster rate of evolution than that of the folded regions; and (2) the disordered regions are less genetically conserved among the orthologs compared to the folded domains [22,23,24]. Although there is no common biological function among the increasing number of IDPs, several IDPs are reported to serve as molecular chaperones, which suppress protein aggregation, solubilize aggregated proteins, and revert misfolded proteins into their active forms [25,26]. Dehydrins, a well-studied example of IDRs, are members of the large class of late embryogenesis abundant (LEA) proteins in plants [27,28,29]. In the past few years, dehydrins have drawn researchers’ attention because of their cryoprotective activity toward proteins and cells [30,31,32]. Recently, Hughes and Graether [33] showed that the genetically conserved region of dehydrins, also known as the YSK_2_ motif, is an IDR. The motif serves as the key segment responsible for its cryoprotective activity. Another example of a cryoprotective IDP is sericin from silkworms [34]. Sericin contains sequences that are rich in serine and glycine; thus, it possesses a highly flexible structure with several random-coil regions. Notably, dehydrins and sericin are not related to each other in terms of their primary sequences. The only known common feature is their disordered regions.

In this study, we hypothesized that IDPs of any sequence may have general cryoprotective activity. Accordingly, we utilized bioinformatics prediction to randomly select IDPs/IDRs from the human genome. We focused on human genome-derived IDPs/IDRs for future biomedical applications as our ultimate goal. The proteins were expressed and IDP/IDR samples were prepared. Next, we examined their cryoprotective activity toward the model enzyme lactate dehydrogenase (LDH). In order to generalize the concept, their cryoprotective activity toward the non-enzymatic protein green fluorescence protein (GFP) was also examined. Finally, lyophilization protection toward LDH was also examined.

## 2. Results

### 2.1. Bioinformatics for Selecting Human Genome-Derived IDPs (Intrinsically Disordered Proteins)

Using the POODLE suites, we chose 53 candidate sequences predicted to be IDPs. Non-redundant proteins or regions of proteins that were predicted to be disordered with high probability were selected from the human protein reference database (HPRD) [35]. Then, candidate sequences were selected based on the following requirements being satisfied: (1) sequences should be a whole protein or a whole domain; (2) sequence length should be shorter than 50 amino acids; (3) for a sequence that covers the whole protein, the probability score of POODLE-W should be >0.6, or POODLE-S should predict >60% of the residues to be disordered; (4) for a sequence that covers an entire domain, POODLE-S should predict >75% of residues to be disordered; and (5) candidate genes are expressed in HeLa cells, or the genes are expressed in mouse and the amino acid sequences of human and mouse orthologs share >99% identity. Sequence redundancy was removed using BLASTClust. Because our purpose was to find novel IDPs, we cross-referenced sequences using DisProt and removed those that were known to be disordered [36]. We cloned 35 out of 53 candidate genes using a standard PCR cloning technique and assessed them by our original thermostable membrane protein-based method [37]. From among them, we randomly selected six potential IDPs (thymosin β10, CstF-77, WWOX isoform3, TNFRSF11B, cortactin isoform a, and transcription elongation regulator 1) and re-cloned them into the IDP-optimized expression system [38]. The sequences are summarized in Table 1. Additional information is also available in Table S1. Note that an additional serine residue was added to the N-terminus of residues 132 to 164 of the transcription elongation regulator 1 (IDP-C1) because of the proteolytic cleavage site of the expression vector [38].

### 2.2. Disordered State of Predicted IDPs/IDRs

We hypothesized that the cryoprotective activity of plant dehydrins can be generalized to other IDPs. To prove this, we obtained samples of IDPs from the genomes of organisms other than higher plants, such as the human genome. Six genes—thymosin β10 (IDP-B3), CstF-77 (IDP-B4), WWOX isoform3 (IDP-C1), TNFRSF11B (IDP-D10), cortactin isoform a (IDP-E1), and transcription regulator 1 (IDP-C9)—were chosen by following the criteria described above. RefSeq-IDs, amino acid sequences, molecular weights, calculated p*I*s, and other parameters are shown in Table 1 and Table S1. None of the five (plus one) IDPs chosen were related in terms of their sequence. POODLE scores for the prediction of order/disorder propensity are shown in Figure S1. In addition, the charge–hydropathy plots (also known as Uversky plots) are shown in Figure S2. All six peptides showed POODLE scores above 0.5 over the entirety of the selected regions, suggesting that these peptides are potential IDPs. Although we planned this experiment carefully, we observed that IDP-C9 contained a folded WW domain and the structure was already in PDB (accession: 2YSI). Thus, we used this sample as an example of a partially folded IDP for further studies.

**Figure 1:**
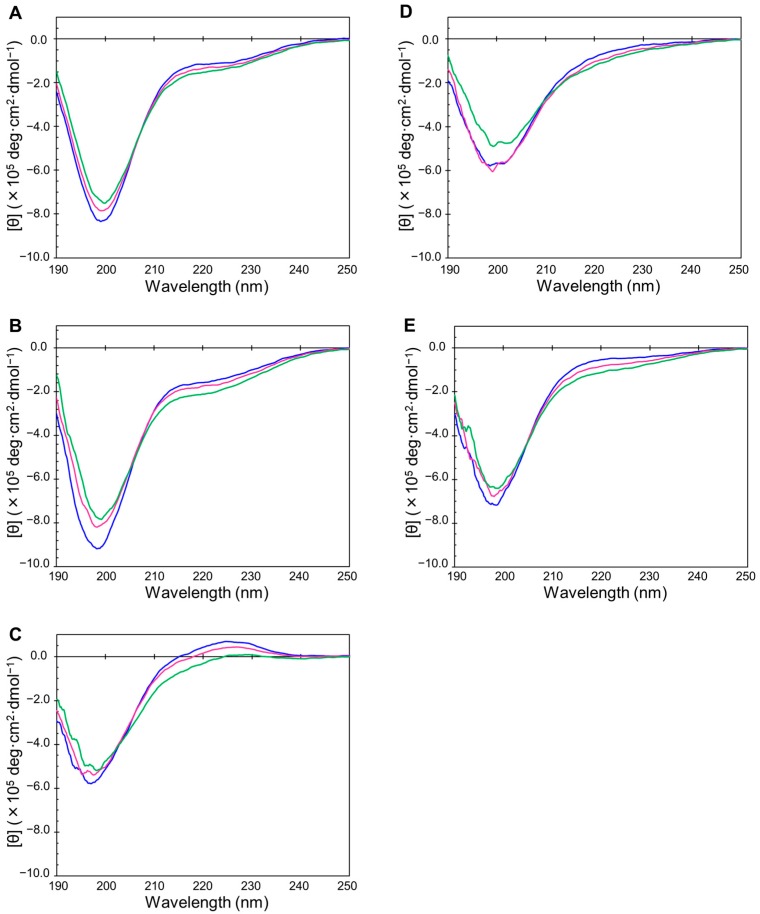
Circular dichroism (CD) spectra of the selected human genome-derived intrinsically disordered protein (IDP) samples. Spectra were measured at 5 °C (navy), 20 °C (red), and 35 °C (green). (**A**) IDP-B3; (**B**) IDP-B4; (**C**) IDP-C1; (**D**) IDP-D10; (**E**) IDP-E1.

**Figure 2:**
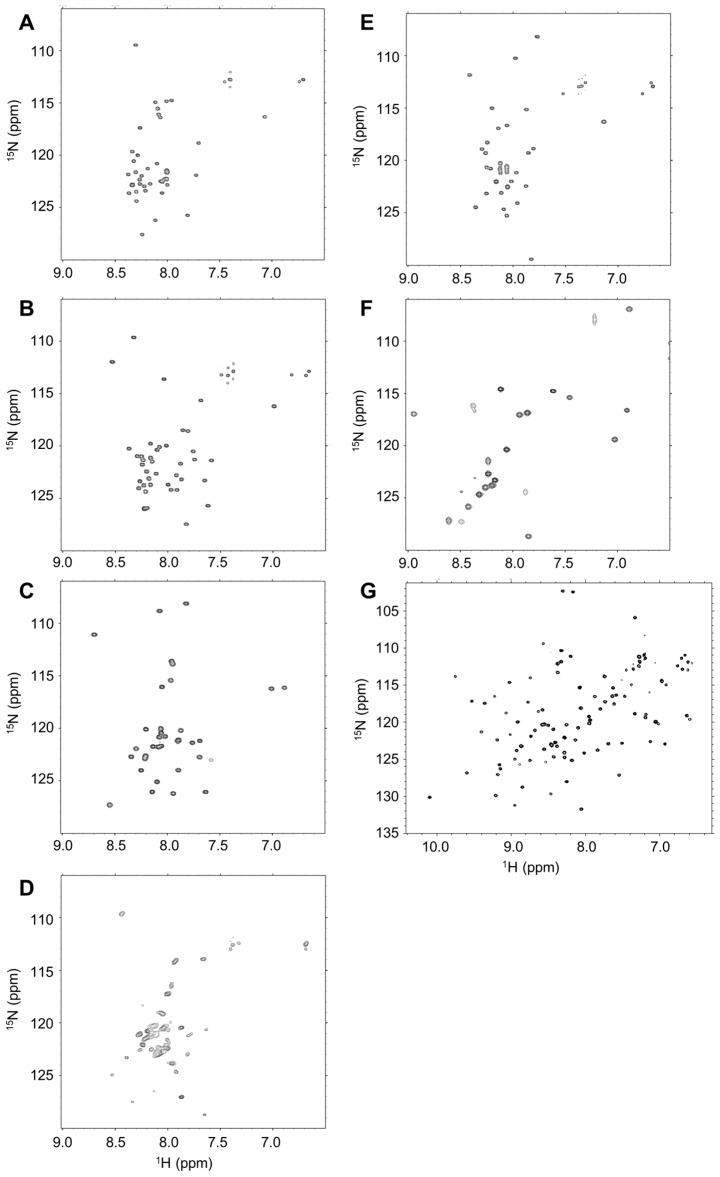
HSQC spectra of the selected human genome-derived IDP samples at 25 °C, pH 6.8. (**A**) IDP-B3; (**B**) IDP-B4; (**C**) IDP-C1; (**D**) IDP-D10; (**E**) IDP-E1; (**F**) IDP-C9 (example of the partly folded peptide); (**G**) mZO1-PDZ1 (example of the completely folded domain).

We then confirmed the disordered state of the selected samples by using circular dichroism (CD), as well as ^1^H–^15^N NMR spectra (Figure 1 and Figure 2). CD and NMR are the most popular methods for assessing whether the sample of interest is an IDP. Generally, an IDP shows a CD spectrum of near-zero Cotton band at 240–210 nm and a negative Cotton band at 200–190 nm. With the exception of IDP-C9, all IDP samples showed typical spectra for random coils from 5–35 °C (Figure 1A–E). In the cases of IDP-B4 and IDP-D10, a small decrease at 200 nm negative Cotton band was observed, which may be due to temperature-induced formation of some structure upon non-specific oligomerization. We did not observe any temperature transition for these five IDPs. This suggests that there is no significant formation of local structures. The first five IDP samples also showed typical HSQC spectra in which the observed ^1^H chemical shift of NH signals showed narrow dispersion between 7.6 and 8.6 ppm (Figure 2A–E) [7,39]. In contrast, the partially folded IDP-C9 (Figure 2F) and the folded mZO1-PDZ1 (Figure 2G) samples gave HSQC spectra of well-dispersed ^1^H chemical shifts of the signals [40]. In Figure 2D, IDP-D10 showed somehow broadened NH signals with several minor peaks. This was probably due to dynamic *cis*-*trans* equilibrium of proline residues in the sequence.

### 2.3. Cryoprotective Activity of Human Genome-Derived IDPs

**Figure 3:**
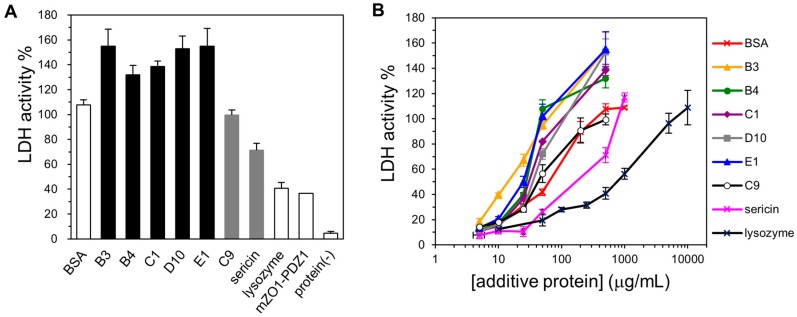
Cryoprotective activity of human genome-derived IDPs and control proteins towards lactate dehydrogenase (LDH). (**A**) LDH activities (final concentration of 50 µg/mL) after freeze-thawing in the presence of 500 μg/mL indicated additive protein. The LDH activity of the untreated sample was set to 100%. IDPs and “partial” IDPs are colored as black and gray, respectively. Protein (-) indicates LDH activity without cryoprotectant; (**B**) LDH activities after freeze-thawing with the given additive protein at various concentrations. Error bars indicate the standard deviation of triplicate measurements.

To examine the cryoprotective activity of the selected human genome-derived IDPs, we chose LDH from rabbit muscle as the model enzyme. Until today, the cryoprotective activity of plant dehydrins [33], silkworm sericin [34], and other low-molecular-weight cryoprotectants [28,41,42,43] have been extensively studied using LDH activity. For controls, we also assayed BSA (a known cryoprotective agent) as a positive control, lysozyme (a poor cryoprotective agent) as a negative control, and mZO1-PDZ1 (an additional example for a folded domain). For the analysis, we set the LDH activity of the untreated sample (the enzyme without freeze and thaw processes and without the addition of a cryoprotectant) as 100%. First, we observed that all five selected IDPs (500 µg/mL) showed increased cryoprotective activity compared to BSA (Figure 3A). Although these proteins have variable sequences, their cryoprotective activity against LDH was quite similar. Second, we observed that the “partial IDP”, IDP-C9, also showed cryoprotective activity. This activity was weaker than that of the “full-IDP”; however, it was still comparable to that of BSA. In contrast, lysozyme and mZO1-PDZ1, both negative controls for a folded protein or protein domain, exhibited lower cryoprotective activity. Moreover, a simple addition of protectants (IDPs and BSA) without freeze-thaw treatment caused a slight (approximately 20%) increase in LDH activity for unknown reasons [44]. We further examined the cryoprotective activity of the IDPs over a concentration range (Figure 3B). The results again showed that the human-derived IDPs were better cryoprotectants than BSA at lower additive concentrations.

**Figure 4:**
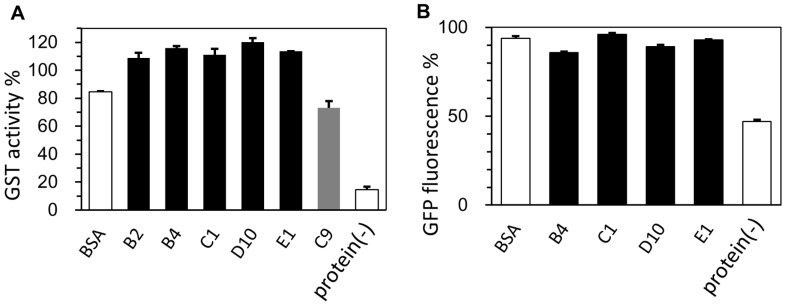
Cryoprotective activity of human genome-derived IDPs and control proteins towards glutathione *S*-transferase (GST) and enhanced green fluorescent protein (EGFP). (**A**) GST activities (final concentration of 11 µg/mL) after freeze-thawing in the presence of 500 μg/mL indicated additive protein. GST activity of the untreated sample was set to 100%; (**B**) EGFP fluorescence (final concentration of 65 µg/mL) after freeze-thawing in the presence of 500 μg/mL indicated additive protein. The EGFP fluorescence of the untreated sample was set to 100%. Error bars indicate the standard deviation of triplicate measurements. IDPs and “partial” IDPs are colored as black and gray, respectively. Protein (-) indicates LDH activity without cryoprotectant.

In order to generalize our observation of the cryoprotective activity of human genome-derived IDPs, Sj26 glutathione *S*-transferase (GST from *Schistosoma japonicum*), as an example of the other enzyme, was assessed (Figure 4A). We found that Sj26 GST is an easily available enzyme that is suitable as a screening system for cryoprotectants. We found that Sj26 GST lost its enzymatic activity to nearly 15% after five times of repeated freeze-thawing. Moreover, as an example of a non-enzymatic protein, enhanced green fluorescent protein (EGFP) from jellyfish was chosen as the sample. In this case, the relative fluorescence intensity of EGFP after ten times of repeated freeze-thawing was monitored with and without cryoprotectants. As expected, since EGFP is suited for fluoroscopic assays, the protein was highly stable and not tending to be inactivated by freeze-thawing processes. After ten times of repeated freeze-thawing, approximately 50% of the fluorescence still remained. Obviously, both of the results were consistent with those of the LDH case, and the human genome-derived IDPs were sufficiently effective as cryoprotectants towards GST and EGFP.

### 2.4. Lyophilization-Protective Activity of Human Genome-Derived IDPs

**Figure 5:**
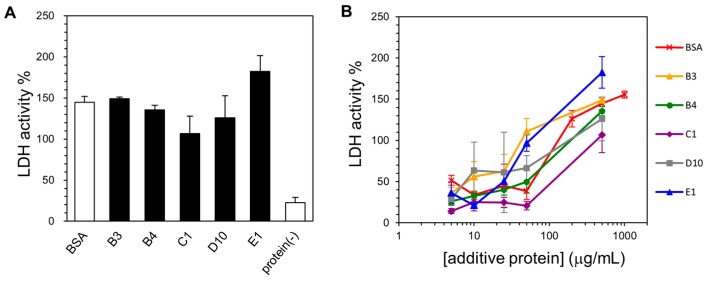
Lyophilization protection of LDH by human genome-derived IDPs and BSA. (**A**) LDH activities (final concentration of 50 µg/mL) after lyophilization in the presence of 500 μg/mL of the given additive protein. The LDH activity of the untreated sample was set to 100%. IDPs are colored as black. Protein (-) indicates LDH activity without lyophilization protectant; (**B**) LDH activities after lyophilization-resolubilizing with the given additive proteins at various concentrations. Error bars indicate the standard deviation of triplicate measurements.

We also assessed the protective activity of IDPs against denaturation during LDH lyophilization. For this purpose, we developed an experimental protocol to assay the lyophilization-protective activity according to the protocol for cryoprotection. We found that LDH lost 90% of its enzymatic activity after one cycle of lyophilization treatment under the given conditions (Figure 5A). We further examined the concentration dependency of lyophilization protection for each IDP (Figure 5B). We found that 500 µg/mL BSA completely protected this loss during lyophilization. Similarly, IDP-B3, IDP-B4, and IDP-E1 showed almost complete protective activity against LDH lyophilization at the same concentration, whereas IDP-B3 and IDP-D10 exhibited slightly less activity. This activity is different from the case of cryoprotection in which all of the selected IDPs exhibited indistinguishable activity. Because lyophilization treatment consists of two processes, freezing and drying, we assumed that the difference in activity among IDPs may have occurred during the drying process. However, here we could not elucidate any explanation based on their amino acid composition.

## 3. Discussion

Our motivation for this study was to answer the following question: “*Are cryoprotection and lyophilization protection general features of IDPs?*” First, Hughes and Graether [33] reported that the genetically conserved core sequence of plant dehydrins, the YSK_2_ motif, is responsible for the cryoprotective activity of dehydrins. They also used NMR to show this segment was an IDP. Based on their observation, we hypothesized that the cryoprotective activity of dehydrin-YSK_2_ is due to its property of intrinsic disorder rather than any unknown “sequence-specific” function. According to this hypothesis, we focused on the human genome-derived IDPs/IDRs because humans are non-hibernating warm-blooded organisms. In this context, it may help in establishing the cryoprotection as an intrinsic property of IDPs/IDRs because no cryoprotection is required in humans. As a result, we showed that at least five unrelated IDPs derived from the human genome exhibited cryoprotective activity toward the model enzyme, LDH, whereas the folded proteins—lysozyme and mZO1-PDZ1—did not. We further demonstrated that this cryoprotective activity was also observed toward another enzyme (GST) and a non-enzymatic protein (GFP). On the other hand, the “partial IDP sample”, IDP-C9, showed limited cryoprotective activity. This suggested that the cryoprotective activity correlates to the proteins’ disordered regions but does not depend on amino acid sequence. In our observation, only some parts of the IDPs are likely to have lyophilization-protective activity. Nevertheless, we still do not rule out that other human genome-derived IDPs may have higher lyophilization-protective activity than that of BSA.

To our knowledge, there is no generally accepted molecular function assigned to IDPs. If the cryoprotective activity is general to IDPs, their physicochemical properties should be related to their mechanisms of action. It has been reported that IDPs can interact with various compounds such as proteins, nucleic acids, phospholipids, metal ions, and other small molecules [3,7]. In some cases, such interactions induce a local 3D structure in the disordered region. In other cases, IDPs may form several semi-stable conformations, even in complex with other molecules. Such variety and flexibility in conformation is the key feature of IDPs [7].

For clarifying the mechanisms of IDPs’ cryoprotective activity, we can refer to the mechanisms of known cryoprotectants such as BSA, which was used as the positive control in this study. Tamiya et al. [42] explained BSA’s cryoprotective action through molecular crowding, which is to reduce the occasion of direct collision among the enzyme molecules. For the in vivo stress-resistant mechanisms of dehydrins, Hughes [45] suggested the following: (1) a weak, non-specific and electrostatic interaction between dehydrin YSK_2_ and LDH is important; (2) dehydrins may interact with and protect membranes from cold and dehydration stresses; and (3) dehydrins can bind water, ions, and nucleic acids. In their interpretation, they did not rule out that specific but not-yet-identified conserved amino acid segment(s) in YSK_2_ may have critical functions in the cryoprotection against LDH [45]. Sericin, another known example of a proteinous cryoprotectant, is also predicted to be an IDP. Sericin contains an extraordinarily high ratio of serine and glycine in their primary structure, which are known as disorder-promoting amino acids [34]. Sericin is believed to act as a cryoprotectant through a mechanism similar to BSA with an additional contribution by hydrophilic amino acids [34].

**Figure 6:**
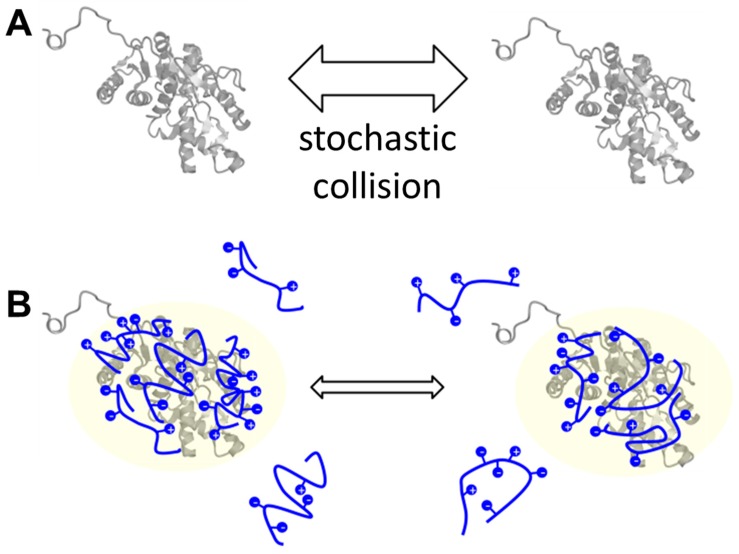
Schematic representation of the cryoprotective/lyophilization protective action of IDPs. The common mechanism of cryoprotection and lyophilization protection is based on the model of molecular shield function proposed by Chakrabortee et al. (see text) [48]. The LDH molecule is represented by the gray ribbon diagram of a protein (representation). Protectants with positive and negative charges are represented as strings. Thick and thin arrows indicate higher and lower rates of direct collision between two enzyme molecules, respectively. (**A**) In the absence of additive protectant molecules, the two enzyme molecules have a certain rate of encounter; (**B**) In the presence of protectants, the stochastic collision rate is decreased, thereby reducing aggregation.

Herein, we discovered five novel IDPs that exhibited cryoprotective activity and whose amino acid sequences are not related to the other known cryoprotectants. Two common features among our IDPs, dehydrins, and sericin are a high content of hydrophilic/charged amino acids and a lack of folded structure. The high content of hydrophilic/charged residues is also an authentic signature of IDPs [2,9]. Recently, some hydrophilic/charged regions in proteins have been found to act as intramolecular chaperones [46,47]. Such chaperone-like anti-aggregation activity is known as “entropic bristle” [46]. Although the hypothesis has good potential, this was not the case for our experiments because we observed that IDPs functioned as additives for cryoprotection. Finally, Chakrabortee et al. [48] reported that IDPs may act as “molecular shields” by weakly surrounding the molecules through non-specific interactions to prevent direct collision of the proteins of interest. Thus, taking into account this knowledge together with our observation, we hypothesize that the human genome-derived IDPs act as “molecular shields” for LDH, thereby exhibiting cryoprotective activity (Figure 6).

Finally, in this study, we demonstrated five new potent cryoprotectants from the human genome, as well as three IDPs (IDP-B3, IDP-B4, and IDP-E1) with lyophilization-protective activity. Such protective properties of IDPs are important not only in basic biophysical science but also for industrial and biomedical applications. The human genome-derived cryoprotective peptides are useful as additives to biomedicines such as therapeutic enzymes, hormones, therapeutic monoclonal antibodies, and other biologics. Because such biomedicines may lose their activity by denaturation and/or aggregation during long-term storage, stability of the proteins during freezing and/or freeze-drying processes is critical. We showed that the human genome-derived IDPs were more potent cryoprotectants than BSA and silkworm sericin. Because of their potential immunological reactions in the human body, both BSA and sericin are not ideal for medical use. Human serum albumin (HSA) might be an alternative if it were cost effective. In contrast, human genome-derived IDPs are expected to be potentially digestive and short-lived [49,50], making them immunologically inert to the human body compared to BSA and other synthetic polymers. In addition, we obtained the potent cryoprotective peptides within a few trials. Thus, we expect that additional peptides between 30 and 50 amino acids in length derived from the intrinsically disordered regions in the human genome may have similar cryoprotective activity. In conclusion, we propose that IDPs could be widely useful as cryoprotectants/lyophilization protectants for enzymes and proteins including biological therapeutics.

## 4. Materials and Methods

### 4.1. Expression and Preparation of the IDP Samples And Non-IDP Proteins

Semi-large-scale preparation of IDP samples was performed as previously described [38]. In brief, the N-terminal autoprotease N(pro) from bovine viral diarrhea virus was selected as a fusion partner for protein expression using the pET-based N(pro) fusion protein expression system [40] optimized for IDP preparation. The IDPs were expressed and the N-terminal tags were removed and finally purified by reversed phase HPLC (COSMOSIL^®^ 5C18-AR-300, Nacalai Tesque, ϕ4.6 mm × 250 mm) with 0.1% trifluoroacetic acid—acetonitrile solvent system. All the peptides were quantified by UV absorbance at 280 nm, lyophilized, and stored at −30 °C until use. As a control for folded proteins and protein domains, we used hen egg lysozyme (Wako, 122-02673) and the first PSD-95/Dlg/ZO-1 (PDZ) domain of recombinant mouse ZO-1 (mZO1-PDZ1) [40].

The active *Schistosoma japonicum* glutathione-S transferase (Sj26 GST) enzyme was expressed in *E. coli* BL21(DE3) harboring pGEX-3T (GE Healthcare Bioscience, Little Chalfont, UK) with an appropriate stop codon. The protein sample was affinity-purified by glutathione-Sepharose^®^ according to the manufacturer’s instruction. The protein sample of EGFP was expressed in *E. coli* BL21(DE3) harboring pET-15b-based plasmid containing a synthetic DNA construct encoding enhanced green fluorescent protein (EGFP) (Genbank:AAB02572) with an appropriate stop codon. This N-terminally His-tagged EGFP was purified by Ni^2+^-affinity chromatography.

### 4.2. Circular Dichroism (CD) Measurements

Circular dichroism CD spectra between 190 and 250 nm were collected on a J-805 spectropolarimeter (JASCO, Tokyo, Japan) at 5, 20, and 35 °C. The time constant, scan speed, bandwidth/resolution, and sensitivity of the spectropolarimeter were set at 1 s, 100 nm/min, 1 nm, and 0.1 deg, respectively. We measured a 300 μL solution of 250 µg/mL IDPs in 10 mM sodium phosphate buffer (pH 7.4) in a quartz cuvette with a 1 mm light path length.

### 4.3. Nuclear Magnetic Resonance (NMR) Analysis

NMR experiments were performed on a Avance III 600 MHz NMR spectrometer (Bruker Biospin, Billerica, MA, USA) equipped with a cryogenic probe and pulsed-field gradients. For the NMR experiments, the final concentration of 0.2-mM [15N] IDP samples were dissolved in 0.3 mL of H_2_O–D_2_O (9:1) containing 25 mM sodium phosphate buffer (pH 6.8) at 25 °C. All two-dimensional spectra were processed with nmrPipe [51] and analyzed with the nmrDraw program. The sample concentrations of the selected IDPs IDP-B3, IDP-B4, IDP-C1, IDP-D10, IDP-E1, and IDP-C9 were 1005, 990, 830, 922, 814, and 798 µg/mL, respectively.

### 4.4. Cryoprotection Assay and Analysis

We selected rabbit muscle lactose dehydrogenase (LDH) (Sigma-Aldrich, St. Louis, MO, USA, L-2500) as a model enzyme for the cryoprotection assay, which was performed with all proteins using a modified technique of Hughes and Graether [33]. BSA (fatty acid-free, Wako, Tokyo, Japan, 013-15143) and silkworm sericin mixture (Pure Sericin, Wako, 167-22681) were selected as positive controls. The initial LDH solution contained 50 µg/mL in 10 mM sodium phosphate (pH 7.4). In a 1.5-mL microfuge tube, we mixed a 10-µL aliquot of the LDH solution with 10 µL solution containing each of the individual protectants (IDPs, BSA, sericin, or the negative control proteins) at the concentrations ranging from 5 to 500 µg/mL. The samples were then frozen in liquid nitrogen for 30 s and thawed in a water bath at 4 °C for 5 min. This cycle was repeated five times. Finally, LDH activity was measured using a standard NADH oxidase coupled-enzyme system. In brief, 3 µL of LDH enzyme solution was diluted with 150 µL of buffer (10 mM sodium phosphate, pH 7.4, 0.2 mM NADH, 1 mM pyruvic acid). NADH oxidation was monitored at Abs340 on a UVmini-1240 spectrophotometer (Shimadzu Co., Ltd., Kyoto, Japan) every 30 s over 3.5 min. All measurements were performed in triplicate.

In addition, we developed the cryoprotection assay for our second model enzyme Sj26 GST. An aliquot of the enzyme (initial 0.8 µM solution) was dialyzed against the buffer containing 0.1 M potassium phosphate (pH 6.5) before use. In a 1.5-mL microfuge tube, we mixed a 25-µL aliquot of the GST solution with 25 µL solution containing each of the individual protectants (IDPs, and sericin) at the concentrations ranging from 5 to 1000 µg/mL. The samples were then frozen in liquid nitrogen for 30 s and thawed in a water bath at 20 °C for 5 min. This cycle was repeated five times. Finally, GST activity was measured using a standard 1-chloro-2,4-dinitrobenzene (CDNB, Sigma-Aldrich, 138630) assay by using a 96-well plate and 2300 EnSpire^TM^ Microplate Reader (Perkin Elmer, Waltham, MA, USA). In brief, a final 1 mM CDNB and glutathione were added to the enzyme solution and the absorbance at 340 nm was monitored. All measurements were performed in triplicate.

We also developed the cryoprotection assay for our model protein EGFP as for demonstration against a non-enzymatic functional protein. EGFP (4.8 µM) was dialyzed against 10 mM sodium phosphate (pH 7.4). In a 1.5-mL microfuge tube, we mixed a 50-µL aliquot of the EGFP solution with 50 µL solution containing each of the individual protectants (IDPs, and BSA) at concentrations ranging from 5 to 1000 µg/mL. The samples were then frozen in liquid nitrogen for 60 s and thawed in a water bath at 25 °C for 5 min. This cycle was repeated ten times. The remaining “activity” of EGFP was estimated by its fluorescence at 511 nm (excited at 460 nm) in a 96-well plate by using a 2300 EnSpire™ Microplate Reader (Perkin Elmer).

### 4.5. Lyophilization Protection Assay and Analysis

A 20-µL aliquot of the same enzyme mixture containing LDH and each of the cryoprotectants was lyophilized. Samples were aliquoted into 1.5-mL microfuge tubes, followed by freezing in liquid nitrogen for 5 min and overnight lyophilization under a vacuum. The lyophilized powder was then re-dissolved in 20 µL of double-distilled water. LDH activity was similarly examined.

## Figures and Tables

**Table 1:** Entry name of the intrinsically disordered protein (IDP) samples in this study, RefSeqID, start and end positions, length, total number of residues of full-length protein, amino acid sequence, and calculated molecular weights are indicated. Start and end indicate the residue numbers corresponding to the full-length protein. M.W. indicates molecular weight. Asterisk indicates an additional serine residue that was added to the N-terminus of C9.

IDP Name	RefSeq ID	Start	End	Length/Total Res.	Sequence	M.W.
B3	NP_066926	1	44	44/44	MADKPDMGEIASFDKAKLKKTETQEKNTLPTKETIEQEKRSEIS	5025
B4	NP_001317	1	44	44/44	MSGDGATEQAAEYVPEKVKKAEKKLEENPYDLDAWSILIREAQV	4951
C1	NP_570859	1	36	36/36	MAALRYAGLDDTDSEDELPPGWEERTTKDGWVYYAK	4150
D10	NP_002537	24	62	39/401	FPPKYLHYDEETSHQLLCDKCPPGTYLKQHCTAKWKTVC	4610
E1	NP_005222	305	342	37/550	GFGGKYGVQKDRMDKNASTFEDVTQVSSAYQKTVPVE	4068
C9*	NP_001035095	132	164	34*/1077	S*PTEEIWVENKTPDGKVYYYNARTRESAWTKPDG	3989

## Supplementary Materials

Supplementary materials can be found at http://www.mdpi.com/1422-0067/19/2/401/s1.
