# Supplementary material for: Discovery of Cryoprotective Activity in Human Genome-Derived Intrinsically Disordered Proteins
Source: Int J Mol Sci. 2018 Jan 30;19(2):401. doi: 10.3390/ijms19020401 (PMC5855623; doi:10.3390/ijms19020401)
Supplement: Supplementary file 1 [file ijms-19-00401-s001.pdf]

Table S1. Entry name of IDP, annotated protein name in RefSeq (source), calculated isoelectric point (pI), number and content of Asp and Glu residues, and number and content of Arg and Lys residues are indicated. pI is calculated by ExPasy ProtParam tool (http://www.expasy.org). Absolute mean net charge and mean scaled hydropathy are calculated by PONDR® (http://www.pondr.com/).

IDP name	source	p*I*	Asp, Glu	Lys, Arg	absolute mean net charge	mean scaled hydropathy
B3	thymosin β10	5.31	10 (22.7%)	9 (20.5%)	0.0227	0.3545
B4	cstF-77	4.53	10 (22.7%)	6 (13.6%)	0.0909	0.4152
C1	WWOX isoform 3	4.16	9 (25.0%)	4 (11.1%)	0.1389	0.3870
D10	TNFRSF11B	7.87	4 (10.3%)	5 (12.8%)	0.0256	0.4108
E1	cortactin isoform a	6.24	5 (13.5%)	5 (13.5%)	0.0000	0.4105
C9	transcription elongation regulator 1	6.56	6 (18.2%)	5 (15.2%)	0.0294	0.3435

International Journal of
Molecular Sciences

MDPI

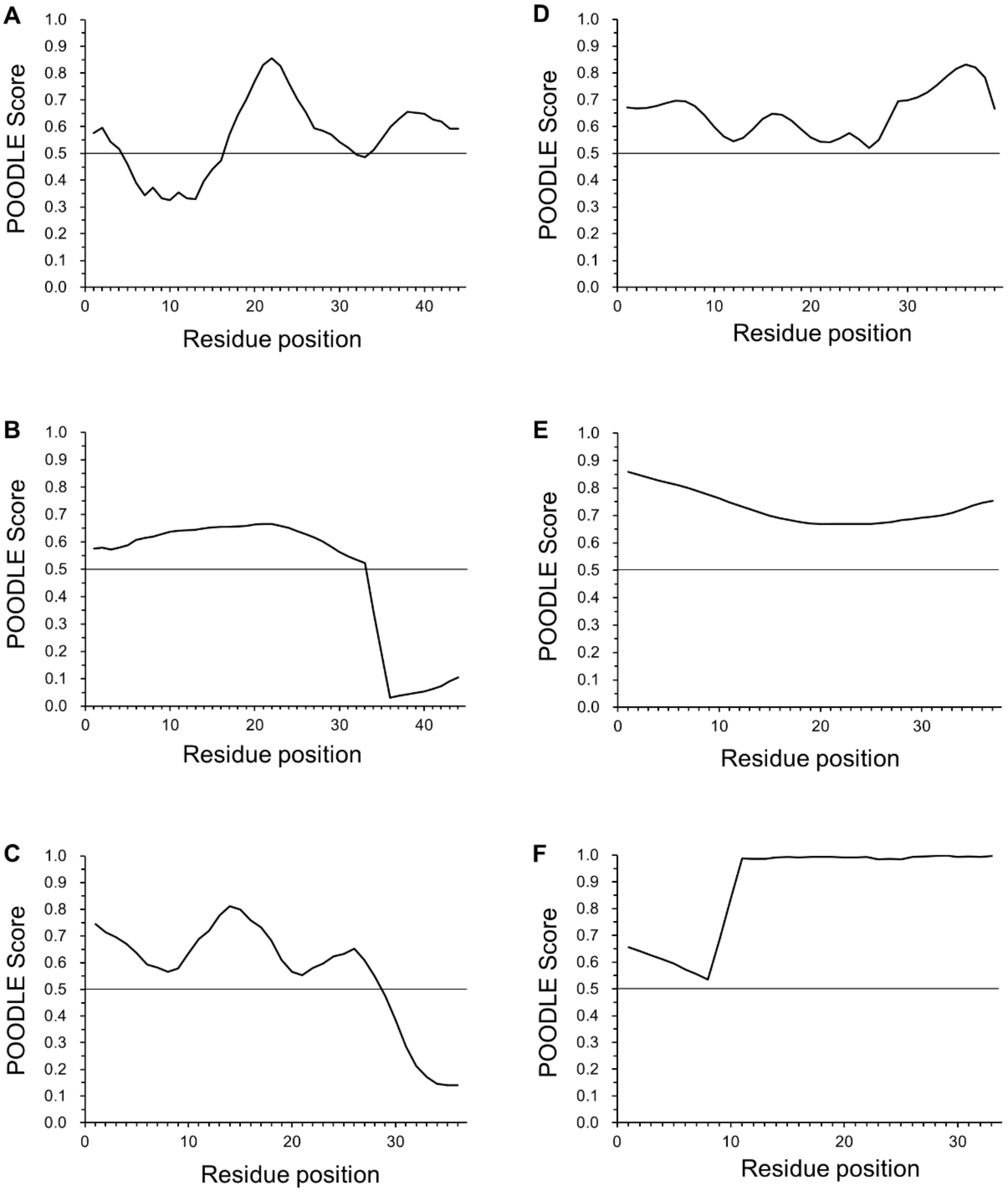

Figure S1. Predicted propensities of protein intrinsic disorder of the selected IDP samples. POODLE scores of the given IDP samples were plotted against residue numbers. Scores >0.5 correspond to disordered residues. (**A**), IDP-B3; (**B**), IDP-B4; (**C**), IDP-C1; (**D**), IDP-D10; (**E**), IDP-E1; (**F**), the partially folded sample IDP-C9.

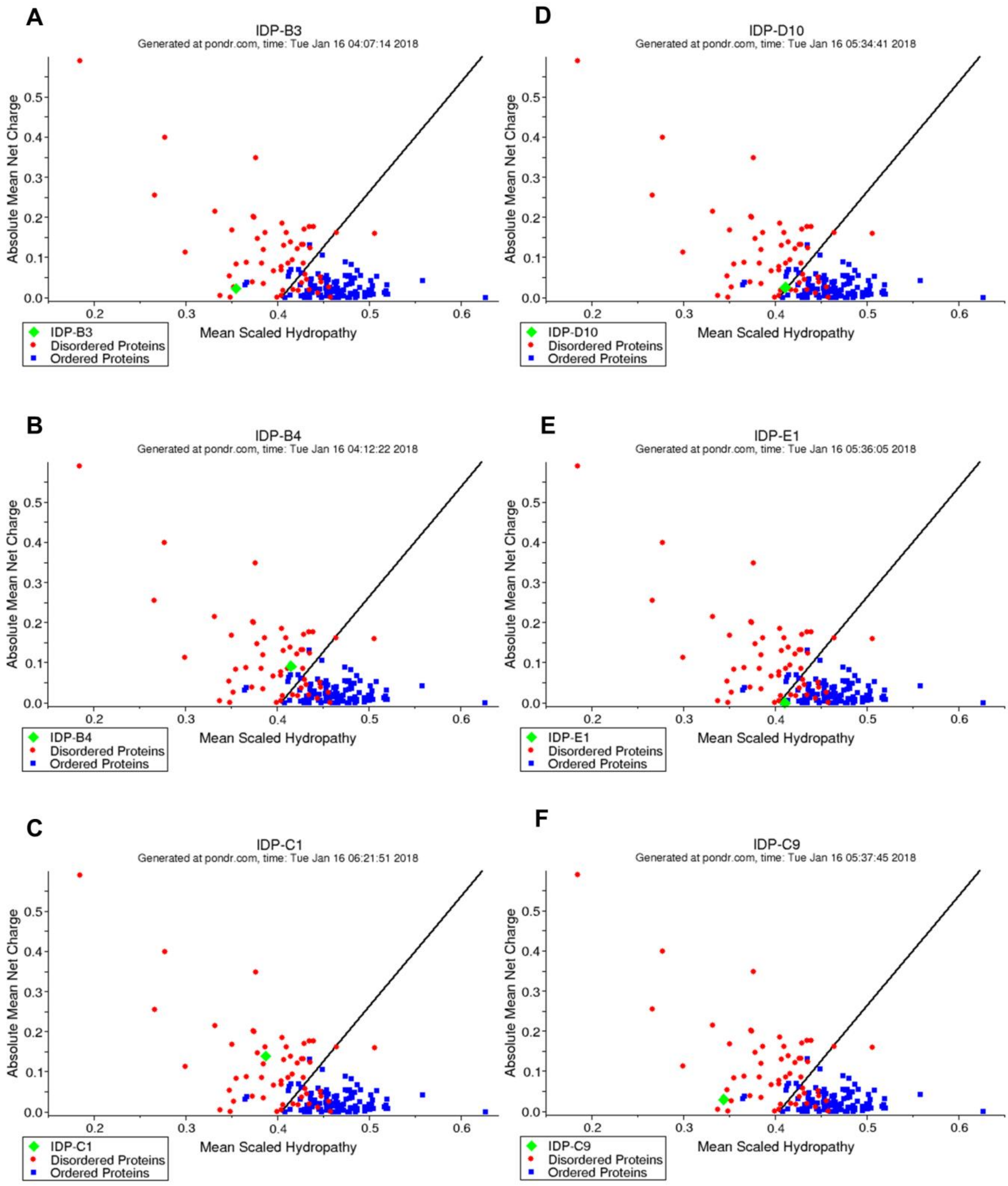

Figure S2. Charge-Hydropathy plots (Uversky plot) of the selected IDP samples. (**A**). IDP-B3; (**B**). IDP-B4; (**C**). IDP-C1; (**D**). IDP-D10; (**E**). IDP-E1; (**F**). the partially folded sample IDP-C9. The figures are generated by PONDR® (http://www.pondr.com/).
